# *De Novo* Transcriptome Assembly, Functional Annotation and SSR Marker Discovery of Qinling Takin (*Budorcas taxicolor bedfordi*)

**DOI:** 10.3390/ani11082366

**Published:** 2021-08-11

**Authors:** Ju Qiu, Rui Guo, Yidan Li, Yuyao Zhang, Kangsheng Jia, Yinghu Lei, Linsen Zan, Anning Li

**Affiliations:** 1 College of Animal Science and Technology, Northwest A&F University, Xianyang 712100, China; qj2403@nwafu.edu.cn (J.Q.); gr11177@nwafu.edu.cn (R.G.); liyidan124@163.com (Y.L.); zhangyuyao@nwafu.edu.cn (Y.Z.); zanlinsen@163.com (L.Z.); 2 Research Center for the Qinling Giant Panda (Shaanxi Rare Wildlife Rescue Base), Shaanxi Academy of Forestry, Xi'an 710402, China; Kangsheng-jia@163.com (K.J.); 8takin@163.com (Y.L.)

**Keywords:** takin, *Budorcas taxicolor bedfordi*, transcriptome, SSR, RNA-Seq

## Abstract

**Simple Summary:**

The golden takin is an endemic ruminant that has been listed as an endangered and vulnerable species by the International Union for Conservation of Nature. Because it lacks a reference genome, little is known about its molecular genetic information. Therefore, this study used RNA sequencing followed by *de novo* assembly, annotation and microsatellites prediction to assess the transcriptome of golden takin muscles. These results provide valuable information for genetic and genomic studies on takin, which will be helpful for protecting the wild takin.

**Abstract:**

The takin (*Budorcas taxicolor*) is an endemic ruminant species belonging to the bovine family. The International Union for Conservation of Nature (IUCN) has listed it as an endangered and vulnerable species. However, little is known about its molecular characterization since it lacks a reference genome. This study used RNA sequencing followed by *de novo* assembly, annotation and simple sequence repeats (SSRs) prediction to assess the transcriptome of Qinling takin (*Budorcas taxicolor bedfordi*) muscles. In total, 21,648 unigenes with an N50 and mean length of 1388 bp and 817 bp, respectively, were successfully detected and annotated against the public databases (NR, GO, KEGG, and EggNOG). Furthermore, 6222 SSRs were identified using the MIcroSAtellite (MISA) identification tool software. Taken together, these findings will provide valuable information for genetic, genomic, and evolutionary studies on takin.

## 1. Introduction

The takin (*Budorcas taxicolor*) is an endangered and endemic species distributed across Asia and is mainly distributed in southwest and northwest China, Nepal, Myanmar, India, and Bhutan [1]. Generally, it is classified into four subspecies based on distinct differences in physical characteristics and geographic location. These include Qinling takin (*B. t. bedfordi*), also known as golden takin, Sichuan takin (*B. t. tibetana*), Bhutan takin (*B. t. whieti*), and Mishmi takin (*B. t. taxicolor*). The average divergence among *B. t. bedfordi*, *B. t. tibetana,* and *B. t. taxicolor* was 1.66% from the D-Loop of mitochondrial DNA (mtDNA) [2]. Among them, the Qinling and Sichuan subspecies are only found in China [3]. Some of the most protected animals in China and the International Union for Conservation of Nature (IUCN) have listed them as endangered and vulnerable species [4].

Based on the comparative morphology, previous studies have reported that takin was closely related to the Musk-Oxen (*Ovibos moschatus*) [5]. However, based on *Cytochrome b* (*Cytb*) or mtDNA sequences, different molecular analyses have suggested that it is closely related to Caprinae [6,7,8,9,10,11,12]. So far, apart from it belonging to the bovine family, its other taxonomic status is still unclear.

In recent years, poaching of takins has occurred frequently. However, it is difficult to distinguish the carcasses of takins from those of oxen and sheep. Therefore, it is necessary to explore the simple sequence repeats (SSRs) markers identifying the carcass of takin at the molecular level so as to help prevent poaching. However, the underlying studies on molecular markers and functional genes have remained largely unknown due to the lack of a reference genome. Because of the deep coverage and single base-pair resolution, RNA sequencing (RNA-Seq) is an efficient method to analyze large-scale transcriptome sequence investigations and obtain molecular markers, even in species lacking a reference genome [13,14]. This next-generation sequencing (NGS)-based technique has recently been widely used for *de novo* transcriptome sequencing assembly, novel gene and molecular marker discovery, and investigating gene expression in various species [15,16,17,18,19]. Therefore, this study used RNA-Seq and *de novo* assembly to characterize and annotate the transcriptome of takin muscle, and several unigenes and microsatellite markers were identified. This information will be helpful to protect the takin through genomic and genetic studies.

## 2. Materials and Methods

### 2.1. Sample Collection and Preparation for Transcriptome Sequencing

The leg muscle was isolated from a male takin (about 16 years old) that died of natural causes at the Rare Wildlife Rescue and Breeding Research Center, and then it was frozen in liquid nitrogen. TRIzol reagent (Invitrogen, Carlsbad, CA, USA) was used to extract total RNA following the specific instructions from the manufacturer. Briefly, mRNA was purified from 5 μg total RNA using poly-T oligo-attached magnetic beads. A total of 300 ± 50 bp cleaved mRNA fragments paired-end were sequenced using the Illumina Novaseq™ 6000 (LC Sciences, Houston, TX, USA) and cloned into paired-end libraries. All other subsequent steps were performed as described previously [20].

### 2.2. De Novo Assembly

Cutadapt 1.9.1 [21] and Perl scripts with default parameters were used to filter high-quality curated data. Firstly, the adapter sequences and poly-A/T were removed. Then, the low-quality and short reads were cleaned out, including reads with “N” more than 5% or reads less than 100 bp in length. FastQC (http://www.bioinformatics.babraham.ac.uk/projects/fastqc/, accessed on 21 June 2020) was used to verify the quality with the Q20, Q30 and GC-content of the clean data. Trinity 2.4.0 was used to reconstruct the transcriptome using default parameters to generate contigs [22]. 

### 2.3. Functional Annotation

All assembled unigenes were aligned against the non-redundant (NR) (http://www.ncbi.nlm.nih.gov/, accessed on 29 June 2020), SwissProt (http://www.expasy.ch/sprot/, accessed on 29 June 2020), and Kyoto Encyclopedia of Genes and Genomes (KEGG) (http://www.genome.jp/kegg/, accessed on 29 June 2020) databases. Blast2GO was performed to analyze the gene ontology (GO) functional classification (http://www.geneontology.org/, accessed on 29 June 2020). To predict possible functions, all assembled unigenes were aligned against the EggNOG (http://eggnogdb.embl.de/, accessed on 29 June 2020) databases. DIAMOND software was used to generate alignments [23]. All other remaining steps were performed as described previously [24]. 

### 2.4. Identification of SSR

The MIcroSAtellite program (MISA, http://pgrc.ipk-gatersleben.de/misa/, accessed on 30 June 2020) [25] was utilized to identify and localize SSR motifs that were screened from mono-nucleotides to hexa-nucleotides with a minimum of five repeats.

### 2.5. Phylogenetic Analysis

The unigene sequences of MYOZ2, MYOZ3, MYL1, TNNC2, RXRG and FAM213A were selected to align with other homologs using BLASTx search program (http://blast.ncbi.nlm.nih.gov/Blast.cgi/, accessed on 14 July 2021). The phylogenetic tree was constructed by a neighbor-joining method with 1000 bootstrap replicates using the MEGA6 program [26].

## 3. Results and Discussion

### 3.1. Sequencing and De Novo Assembly of Takin Transcriptome

In total, 56,745,076 raw reads were generated from the Illumina Novaseq™ 6000 platform. After trimming the adapters and filtering out the low-quality and short reads, 55,417,300 clean and high-quality reads were produced. This sequence quality analysis had a Q30 and GC percentage of 94.43% and 53.13%, respectively (Table 1). Validation studies suggested that a high Q30 value correlates with high-quality sequencing data and could be used for transcriptome assembly. These clean reads were assembled into 25,677 transcripts that had an N50 of 1596 bp and average length of 925 bp, respectively. Moreover, 21,648 unigenes had an N50 and mean length of 1388 bp and 817 bp, respectively (Table 1).

**Figure 1:**
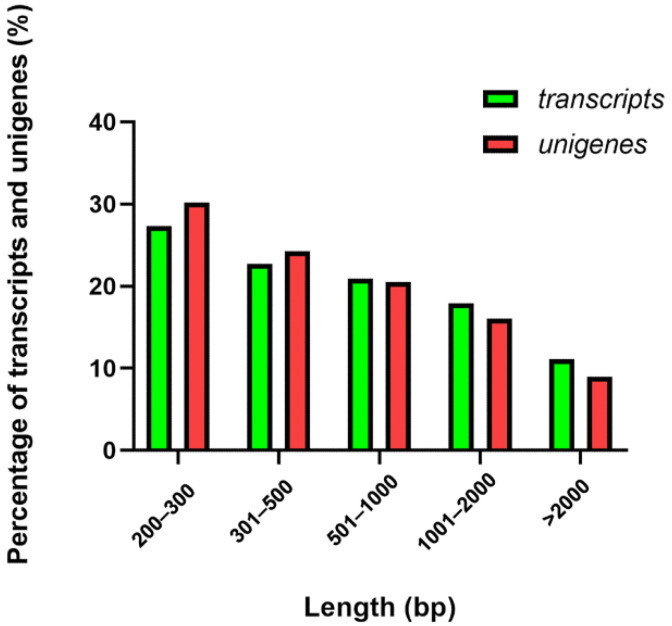
The length distribution of transcripts and unigenes.

The length and distribution analysis of assembled transcripts and unigenes revealed that most of them were longer than 300 bp. Among these transcripts, 27.34% of the assembled sequences were shorter than 300 bp, 22.71% ranged from 301 to 500 bp, 20.95% varied from 501 to 1,000 bp, 17.89% scaled from 1001 to 2000 bp, and 11.10% were longer than 2000 bp (Figure 1). Among the unigenes, 30.21% of the assembled sequences were less than 300 bp, 24.26% varied from 301 to 500 bp, 20.55% ranged from 501 to 1000 bp, 16% scaled from 1001 to 2000 bp and the remaining 8.97% were more than 2000 bp (Figure 1).

Compared to a mean length of 863 bp and a Q20 of 97.6% from sheep muscle [27], the average length was longer and the value of Q20 is larger in this study. Compared to an average length of 972.41 bp, an N50 of 1,505 bp and 55.72% with longer than 500 bp in length from Chinese Swamp Buffalo [19], the value was slightly lower in this study.

### 3.2. Functional Annotation

Table 2 summarizes the assembled unigenes sequences aligned against public protein and nucleotide databases, which are based on sequence similarities to the published protein and nucleotide databases, respectively. Among the 21,648 unigenes, 13,214 (61.04%), 12,071 (55.76%), 10,329 (47.71%), 12,157 (56.16%), 11,606 (53.61%) and 8890 (41.07%) could be matched in the NR, GO, KEGG, EggNOG, SwissProt and Pfam databases, respectively.

#### 3.2.1. NR Annotation

**Figure 2:**
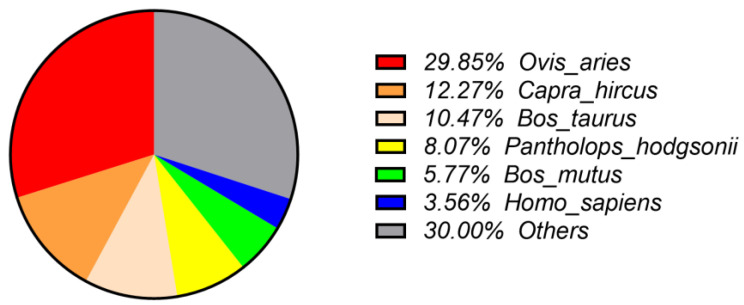
Similarity analysis of unigenes based on the best hit.

Based on BLASTx similarity analysis results on the unigenes, species distribution showed that the unigenes of *Budorcas taxicolor* had the highest homology to *Ovis aries* (29.85%). It was followed by *Capra hircus* (12.27%), *Bos taurus* (10.47%), *Pantholops hodgsonii* (8.07%), *Bos mutus* (5.77%) and then *Homo sapiens* (3.56%) (Figure 2), which was consistent with the findings from previous studies [8,9,28].

#### 3.2.2. GO Annotation

**Figure 3:**
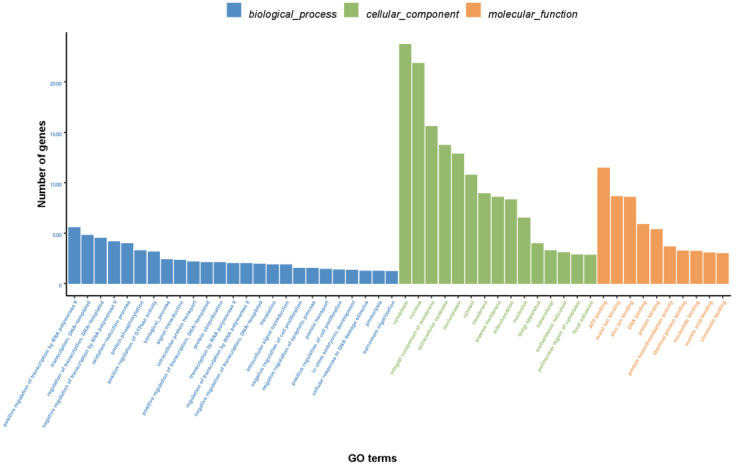
Gene ontology classification of assembled unigenes.

Based on the NR annotation, we used the Blast2GO software to classify 12,071 unigenes into three main Gene Ontology (GO) categories and 50 subcategories (Figure 3). In the biological processes, the top ten GO terms were “positive regulation of transcription by RNA polymerase Ⅱ”, “transcription, DNA template”, “regulation of transcription, DNA templated”, “negative regulation of transcription by RNA polymerase Ⅱ”, “oxidation-reduction process”, “protein phosphorylation”, “positive regulation of GTPase activity”, “biological process”, “signal transduction” and “intracellular protein transport”. On the other hand, in the cellular components, the top five GO terms were “cytoplasm”, “nucleus”, “integral component of membrane”, “extracellular exosome” and “nucleoplasm”. Lastly, the top five GO terms in molecular functions were “ATP binding”, “metal ion binding”, “zinc ion binding”, “DNA binding” and “protein binding” (Figure 3).

#### 3.2.3. KEGG Pathway

**Figure 4:**
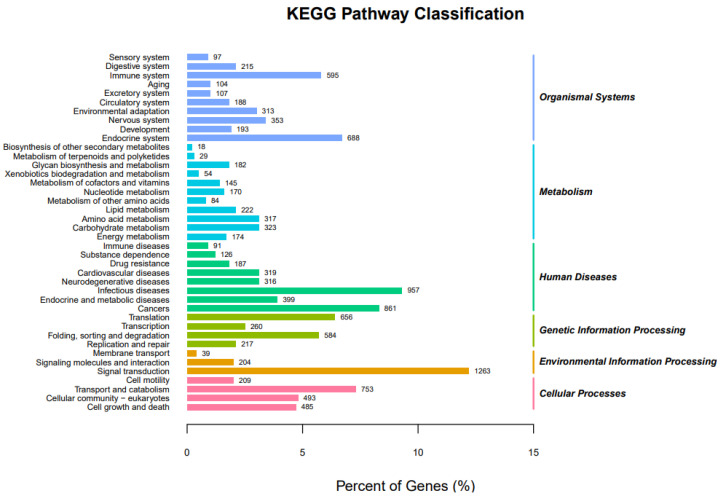
Pathway assignment based on the KEGG.

After the assembled 21,648 unigenes were searched against the KEGG database, 10,329 unigenes were assigned to six main ontologies and classified into 40 pathways (Figure 4). Based on environmental information processing, the top ten enriched pathways were classified into the following categories: signal transduction (1263), followed by human infectious diseases (957), cancers (861) from human diseases, transport and catabolism (753) from cellular processes, endocrine system (688) from organismal systems, translation (656) from genetic information processing, immune system (595) from organismal systems, folding, sorting, and degradation (584) from genetic information processing, cellular community-eukaryotes (493) from cellular processes, and cell growth and death (485) from cellular processes (Figure 4).

#### 3.2.4. EggNOG Classification 

**Figure 5:**
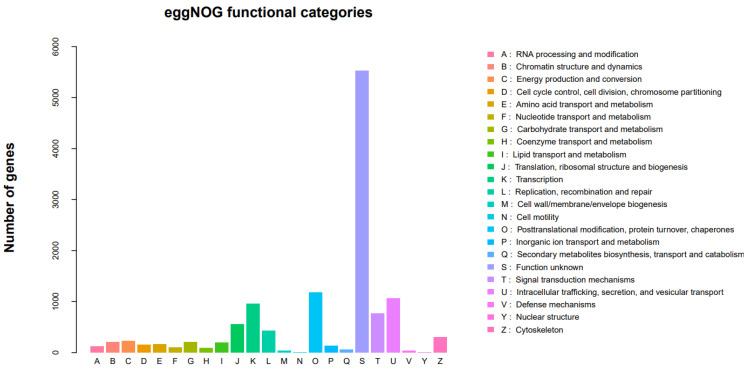
Histogram presentation of EggNOG classification.

In total, 12,157 unigenes were annotated in the EggNOG database via domain-based alignments and classified into 23 functional categories (Figure 5). The largest annotated group was “function unknown”, followed by “posttranslational modification, protein turnover, chaperones”, “intracellular trafficking, secretion, and vesicular transport”, “transcription”, “signal transduction mechanisms”, etc.

### 3.3. Identification of SSR Markers

**Figure 6:**
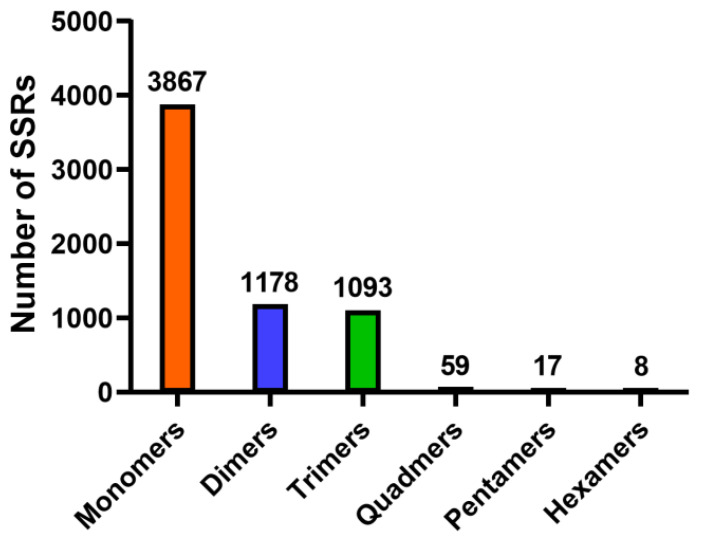
Distribution of SSRs based on the number of repeat units.

Simple sequence repeats (SSRs), also known as microsatellites, are useful molecular markers for population genetics [29]. This study identified 6222 SSRs in 5729 unigenes using the MISA software. Figure 6 shows the distribution and frequencies of SSRs with a minimum of five repeats. The largest proportion was monomers (3867, 62.2%), followed by dimers (1178, 18.9%), trimers (1093, 17.6%), quadmers (59, 0.9%), pentamers (17, 0.3%) and hexamers (8, 0.1%) (Figure 6). Recent studies have reported that SSRs are useful during genetic assessment of various wild animals [30,31,32,33]. To the best of our knowledge, this is the first report that has identified microsatellites in takin. We intend to carry out the *de novo* genome sequencing of takin in the future to identify new SSRs markers that will help evaluate the genetic diversity of takin.

### 3.4. Phylogenetic Analysis

**Figure 7:**
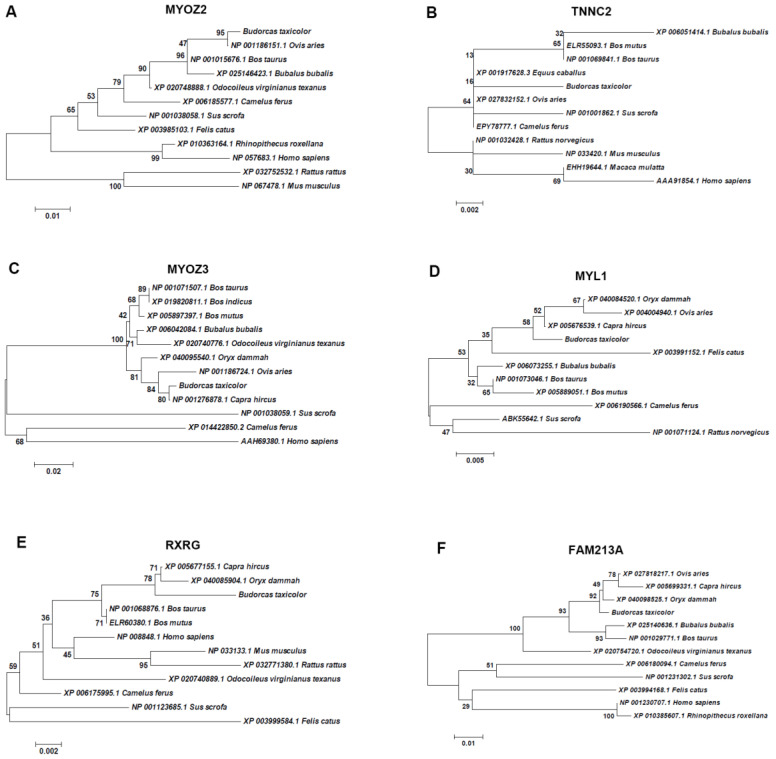
The phylogenetic analysis of assembled unigenes encoding. MYOZ2 (**A**), TNNC2 (**B**), MYOZ3 (**C**), MYL1 (**D**), RXRG (**E**) and FAM213A (**F**) were selected to construct phylogenetic trees. A statistical method used was neighbor-joining with a number of bootstrap replications of 1000. Branch numbers represent bootstrap values.

We selected six unigenes with complete CDS to construct the phylogenetic tree. The homologs of takin were most closely related to sheep, and less so to mouse and human for MYOZ2 (Figure 7A) and TNNC2 (Figure 7B). However, the goat MYOZ2 and TNNC2 homologs were not identified because they were not found in the BLASTx search results. MYOZ3 (Figure 7C) and MYL1 (Figure 7D) homologs of takin were most closely related to goat, followed by sheep, and less so to human and pig, respectively. Further, the RXRG homolog of takin were most closely related to goat and oryx, and less so to pig and cat (Figure 7E). The FAM213A (Figure 7F) homolog of takin was most closely related to oryx, followed by goat and sheep, and less so to human and golden monkey. For the *Cytb* gene sequence, it was found that the homolog of takin was the most closely related to sheep, followed by goat [8,9]. However, the homolog of takin was the most closely related to goat, followed by sheep for the mitochondrial genome sequence [10,11,12]. To reveal the taxonomic status of takins, further research, particularly genome-wide analysis, will be necessary.

## 4. Conclusions

In summary, the high quality of Qinling takin (*Budorcas taxicolor bedfordi*) muscle transcriptome was *de novo* assembled for the first time. A total of 21,648 unigenes were successfully detected and annotated. In addition, 6222 SSRs were identified as putative molecular markers that can help assess genetic diversity. In brief, these results provide valuable information for genetic and genomic studies to protect takins.

## Figures and Tables

**Table 1:** Summary of sequencing data.

Category	Number/Length
Raw reads	56,745,076
Raw bases (Gb)	8.51
Clean reads	55,417,300
Clean bases (Gb)	7.76
Q20 percentage (%)	98.39
Q30 percentage (%)	94.43
GC percentage (%)	53.13
Transcripts	25,677
N50 length (bp)	1596
Mean length (bp)	925
Unigenes	21,648
N50 length (bp)	1388
Mean length (bp)	817

**Table 2:** Functional annotation.

Database	Number of Unigenes	Ratio (%)
All	21,648	100.00
NR	13,214	61.04
GO	12,071	55.76
KEGG	10,329	47.71
EggNOG	12,157	56.16
Swissprot	11,606	53.61
Pfam	8890	41.07

## Data Availability

The data presented in this study are available in the Sequence Read Archive (SRA) database, accession number: PRJNA720167.
